# Unraveling the dark matter, long non-coding RNAs, in male reproductive diseases: A narrative review

**DOI:** 10.18502/ijrm.v13i11.7959

**Published:** 2020-11-22

**Authors:** Masoud Dehghan Tezerjani, Seyed Mehdi Kalantar

**Affiliations:** ^1^Abortion Research Centre, Yazd Reproductive Sciences Institute, Shahid Sadoughi University of Medical Science, Yazd, Iran.; ^2^Department of Genetics, Shahid Sadoughi University of Medical Sciences, Yazd, Iran.

**Keywords:** Long noncoding RNA, cancer, Prostatic hyperplasia, Prostatitis, Varicocele, Sperm abnormalities.

## Abstract

Recent advances in human transcriptome have revealed the fundamental and functional roles of long non-coding RNA in the susceptibility to diverse diseases and pathological conditions. They participate in wide range of biological processes such as the modulating of chromatin structure, transcription, translation, and post-translation modification. In addition, based on their unique expression profiles and their association with clinical abnormalities such as those of related to male reproductive diseases, they can be used to develop therapeutic methods and biomarkers for screening of the diseases. In this study, we will review the identified lncRNAs and their molecular functions in the pathogenesis of male reproductive diseases such as prostate cancer, benign prostatic hyperplasia, prostatitis, testicular cancer, varicocele, and sperm abnormalities.

## 1. Introduction

The prevalence of infertility is 15% worldwide, and according to the global data, 20-70% of the total infertility cases is attributed to male infertility (1). Male infertility is characterized by heterogeneous and multifactorial conditions among which genetics factors encompass approximately 40% of cases with idiopathic infertility (2). The most common clinical abnormalities associated with male infertility are prostate cancer (PCa), prostatitis, benign prostatic hyperplasia (BPH), testicular cancer (TCa), varicocele, and inability to produce normal sperm (azoospermia, oligozoospermia, asthenozoospermia (AZS), and teratozoospermia). Although the main molecular mechanisms involved in the pathogenesis of these abnormalities are unknown, all of them have genetic background (3-6).

**Figure 1:**
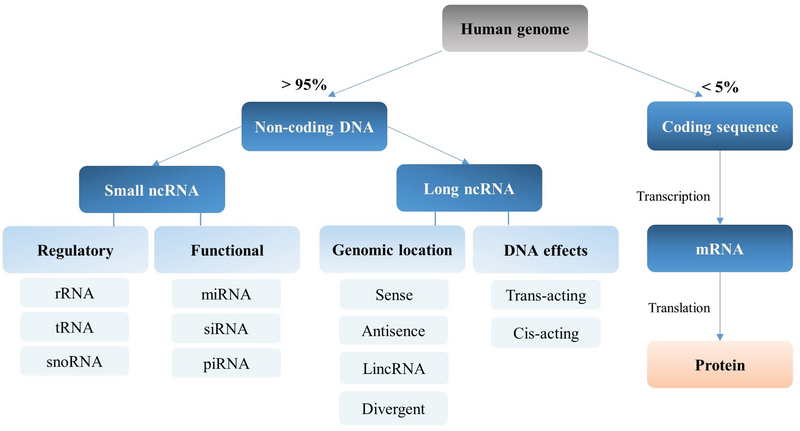
The components of the human genome. Less than 5% of the genome consists of coding sequences, which can ultimately be translated to proteins. More than 95% of genome encompasses non-coding DNA, which is transcribed into two broad groups including small ncRNA and lncRNA. Small ncRNAs are categorized into regulatory (rRNA: 121-5070 nts, tRNA: 73-93nts, snoRNA: 70-200 nts) and functional RNAs (miRNA: 21-25 nts, siRNA: 20-25 nts, piRNA: 24-31 nts). LncRNAs can act as cis-acting lncRNA (affect nearby genes in same chromosome) or trans-acting lncRNA (affect distant gene on other chromosomes). LncRNA can originate from sense, antisense strand, intergenic (lincRNA), and opposite direction to nearby protein (divergent).

**Figure 2:**
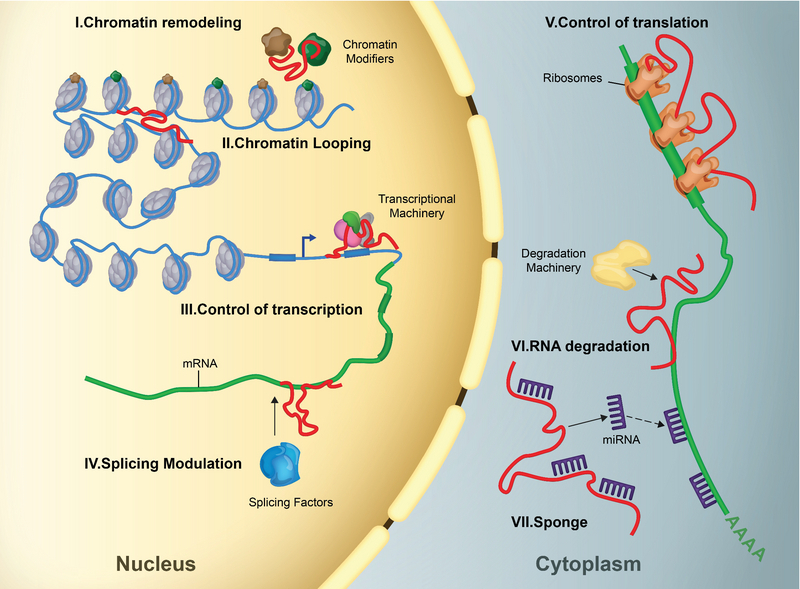
Functions of lncRNAs, (I) lncRNAs (in red) recruit chromatin modifiers such as activator (brown) or repressive (green) Histon remarks to change chromatin organizational pattern. (II) They can act as a structure to create chromatin looping through which, cis-regulatory sequences such as enhancers and silencers interact with target promoters. (III) LncRNAs control transcription by interacting with transcriptional activators and repressors. (IV) LncRNAs can modulate splicing pattern of RNA by interacting with splicing factors and junctions. (V) LncRNAs control translation by inhibiting or favoring the loading of polysome to mRNAs. (VI) LncRNAs protect RNA from degradation or subject them to degradation machinery. (VII) LncRNAs also act as molecular sponge by interacting with their complementary miRNA. They can also occupy their binding sites on RNA molecules.

While more than 85% of the human genome is transcribed, only a low proportion of these transcribed RNAs encode proteins (7). Non-coding RNAs (ncRNAs) are categorized into two broad groups based on their size. Short ncRNAs are < 200 nucleotides (nts) in length and include microRNAs (miRNAs), piwi-interacting RNAs (piRNAs), and small nuclear RNAs (snoRNA) (8), while long ncRNAs (lncRNAs) are > 200 nts in length (Figure 1). lncRNAs are involved in several mechanisms such as chromatin remodeling, chromatin looping, recruitment of transcription factors. They can also be involved in RNA splicing and control of translation. Furthermore, they can also modulate RNA degradation as well as act as a miRNA sponge and sequester them to control gene expression (Figure 2) (9).

The gene regulatory process can occur at any stage such as RNA transcription, translation, and post-translational modification. This process includes DNA methylation, histone modification (methylation, phosphorylation, ubiquitylation, acetylation, and sumoylation) and tissue-specific transcription factors (TFs) (10, 11). In addition, ncRNAs have also been identified as prominent regulatory elements in gene expression (12). The dark matter of the genome, lncRNAs, was previously considered as transcriptional noise (13). However, advances in genomic technologies such as high-throughput sequencing technologies have led to the identification of thousands of lncRNA in different diseases including male reproductive ones. LncRNAs are involved in differentiation, proliferation, and self-renewal of spermatogonial stem cells (SSCs) (14). Furthermore, they modulate apoptosis, invasion, cell cycle, and cell signaling pathways in the male reproductive tract. Therefore, LncRNAs could be considered as a potential biomarker to detect any abnormalities in male reproductive tract. In this review, we will provide a short summary about the role of lncRNAs and their pathways in the pathogenesis of male reproductive-associated diseases such as PCa, prostatitis, BPH, TCa, varicocele, and sperm abnormalities (azoospermia, oligozoospermia, asthenozoospermia, and teratozoospermia).

## 2. Materials and Methods

The current narrative review study aimed to provide the latest information about the lncRNAs involved in the susceptibility to different male reproductive diseases and summarizing lncRNAs functions in the development of the related diseases. It was conducted through a comprehensive search in electronic databases, including PubMed, Scopus, and Google Scholar using Keywords such as lncRNA, prostate cancer, prostatic hyperplasia, prostatitis, testicular cancer, varicocele, sperm abnormalities. After evaluating 122 related articles including original, review, and meta-analysis, 93 articles were finally used in this study.

## 3. Diseases

### Prostate cancer

PCa as a complex and noncutaneous malignancy is the second cause of cancer-associated death among men (15). Several studies have implicated the role of differentially expressed lncRNAs affecting pathological, treatment, and prognosis of PCa. In addition, lncRNAs can act as oncogene and tumor suppressor molecules in PCa tumorigenesis (16, 17). We will discuss some principal oncogenic lncRNAs later in the article. Table I mentions the further oncogenic lncRNAs with their summary of functions.

**Table 1:** Other* known oncogenic lncRNAs involved in PCa


**IncRNA**	**Genomic location**	**Summary of functions**	**Reference**
**SAP30L-AS1**	5q33.2	Binds to promoter of SAP30L and represses it. It promotes PCa cell proliferation and inhibits apoptosis	(18)
**FALEC**	1q21.2	Up regulated in hypoxic environment. It promotes invasion, proliferation and migration in vitro	(19)
**MYU/VPS9D1-AS1**	16q24.3	Promotes expression of c-Myc by competitive binding to mir-184. It induces proliferation of PCa	(20)
**PCSEAT/PRCAT38**	21q22.3	By acting as a sponge for miRNA-143-3p and miRNA-24-2-5p, regulates EZH2. It increases the motility and growth of PCa	(21)
**FOXC2-AS1**	16q24.1	Targets 3'UTR of miR-1253 and binds to complementary site in EZH2. It promotes cell proliferation and PCa growth in vivo and in vitro	(22)
**LINC01116**	2q31.1	Knock down of this lncRNA with siRNA increases the expression of genes such as GAPDH (regulates glycolysis), MAP1LC3B2 (autophagy) and H2AFY (chromatin structure). It increases cell proliferation of PCa	(23)
**LOC400891**	22q11.2	Up regulated in PCa tissue. It is associated with proliferation, migration and invasion in PCa tissues and independent predictor of biochemical free-survival	(24)
**ncRNA-ROR**	18q21.31	Acts as ceRNA and Competes with miR-145 binding in HuPCaSCs (human prostate cancer stem cells), miR-145 decreases cell proliferation in PCa by suppressing Oct4 expression. It promotes cell proliferation in PCa	(25)
**PCA3/DD3**	9q21-22	Knock down of this lncRNA in LNCap cells regulates genes encoding AR cofactors and EMT marker. It increases viability of PCa cells	(26)
**SPRY4-IT1**	5q31.3	Highly upregulated in PCa. Knockdown of this lncRNA in PC3 cells inhibits cell proliferation and invasion of cells	(27)
**LOC440040**	11p11.12	Upregulated in PCa tissues, high expression of this lncRNA is associated with advanced clinical features and shorter overall survival. Its expression is independent prognostic factor in patients	(28)
**ZEB1-AS1**	10p11.22	Recruits methyltransferase MLL1 to promoter of ZEB1 and increases expression of ZEB1 by inducing H3K4me3. It increases proliferation and migration of PCa cells	(29)
**TRPM2-AS**	21q22.3	Overexpressed in PCa. In addition, it regulates TRPM2 gene and genes related to cell cycle and survival such as FYN and AKT1	(30, 31)
*Refers to lncRNAs which are not mentioned in the main text Abbreviations: SAP30L: 30-kDa Sin3-associated Protein, EZH2: Enhancer of zeste homolog 2, UTR: Untranslated region, siRNA: Small interfering RNA, GAPDH: Glyceraldehyde 3-phosphate dehydrogenase, MAP1LC3B2: Microtubule-associated proteins 1A/1B light chain 3 beta 2, H2AFY: H2A histone family member Y, HuPCaSCs: Human prostate cancer stem cells, LNCap: Lymph node carcinoma of the prostate, AR: Androgen receptor, EMT: epithelial-mesenchymal transition, MLL1: myeloid/lymphoid or mixed-lineage leukemia 1, ZEB1: Zinc finger E-box binding homeobox 1 TRPM2: Transient receptor potential cation channel subfamily M member 2			

#### Oncogenic lncRNAs in PCa

Prsenter and colleagues identified 121 unannotated ncRNA transcript in a cohort of 102 prostate tissues and cell lines using high-throughput RNA sequencing and named them as prostate cancer-associated ncRNA transcripts (PCATs) based on their fold-change in PCa samples compared to normal tissue. PCAT-1, ∼ 1.9 kb lncRNA, is located upstream of the c-Myc gene at chromosome 8q24 (32). Its expression is significantly high and upregulated in most PCa cells especially metastatic samples; furthermore, it is mostly found in cytoplasm and in a small amount in the nucleus of PCa cells. It has also been reported that lncRNA PCAT-1 represses BRCA2 involved in homologous recombination (HR), resulting in unrepaired Double-stranded DNA breaks (DSBs) (33). Another study revealed that PCAT-1 with microRNAs (miR-3667-3p and miR-34a) upregulates the c-Myc protein level, which is essential for cell cycle progression, leading to proliferation of prostate tumor cell (34). PCAT-5 is another oncogenic lncRNA that is regulated by transcription factor ERG (ETS-related gene) and is significantly associated with cell proliferation and invasion of PCa cells. In addition, it is upregulated in castration-resistant prostate cancer (CRPC) tissue compared to normal prostate cells (35). The lncRNA Prostate Ovary Testis Expressed Family member Antisense 1 (POTEF-AS1) is the androgen-dependent regulator that is involved in apoptosis and toll-like receptor signaling pathways by interacting with TLR3 and TNFSF10. In addition, it has a fundamental role in the progression of docetaxel-treated cells by repressing apoptosis, resulting in chemoresistance (36).

HOXD antisense growth-associated lncRNA (HOXD-AS1) is upregulated in PCa. A study has reported that HOXD-AS1 regulated the expression of target genes and promoted cell proliferation by recruiting WDR5. This molecule is part of the MLL1/MLL complex and has a key role in histone H3 lysine 4 tri-methylation (H3K4me3) which is associated with transcriptional activation (37). Li and coworkers in 2017 reported that small nucleolar RNA host gene 1 (SNHG1) lncRNA promotes cell proliferation and is upregulated in PCa. By acting as ceRNA (competing endogenous RNA), this lncRNA suppresses the activity of miR-199a-3p that promotes the expression of its target, CDK7, resulting in increased cell proliferation and cell cycle progress in PCa (38). SOCS2-AS1 (Cytokine signaling 2-antisense transcript 1) is an androgen-induced lncRNA and regulates genes TNFSF10, FOXM1, and CENPF, which are involved in apoptosis and cellular proliferation in PCa. Moreover, by repressing apoptosis, it plays a fundamental role in the progression of CRPC (39).

CTBP1-AS, an androgen-responsive lncRNA, is located in the AS region of C-terminal-binding protein 1, which works as a corepressor for the androgen receptor. By recruiting the RNA-binding transcriptional repressor PSF and HDAC (Histon deacetylase), this lncRNA can repress the expression of the CTBP1 gene. Low expression of this gene is associated with overexpression of androgen-related genes and aberrant cell proliferation in prostate cells which causes PCa (40). PVT1 (Plasmacytoma Variant Translocation 1) lncRNA was identified to be associated with miR-146a expression. This lncRNA decreases the miR-146a expression by increasing methylation in CpG Island in the promoter of miR-146a. The silencing of miR-146a suppresses apoptosis and promotes cell viability in PCa (41). Another study by Yang and co-authors revealed that the level of PVT1 expression was notably high in PCa compared to normal prostate cells. In addition, they did knockdown this lncRNA in PCa cell lines and found the expression of cleaved caspase-3 and c-Myc was upregulated and downregulated, respectively. Therefore, this lncRNA increases cell growth in PCa in vitro and in vivo (42).

The gene encoding TTTY15 (Testis-specific transcript Y-linked 15) lncRNA is located at Yq11.2. Some studies revealed that the fusion of the TTTY15 gene with USP9Y (Ubiquitin Specific Peptidase 9 Y-linked) gene is a potential carcinogen in some cancers particularly in PCa (43, 44). Xia and colelagues found that this lncRNA is upregulated in patients with PCa compared to normal cases. They used the CRISPR-Cas9 technique to knock down this lncRNA and reached the conclusion that it suppressed the growth of PCa cells in vitro and in vivo. Moreover, TTTY15 increases CDK6 and FN1 expression by acting as a sponge for microRNA let-7 (45). A single study reported that the expression level of THBS4-003 (Thrombospondin 4) is significantly higher in PCa cells compared to non-tumor ones. It also revealed that knockdown of this lncRNA suppressed the invasive and migratory capability of PCa cell, the expression level of MMP-9 (matrix metalloproteinase-9) as well as p38 (3). Further, Jiang and colelagues found that lnc-MX1-1 (MX Dynamin like GTPase 1) is upregulated in PCa cells compared to adjacent normal prostate cells using array expression profiling. By using RNAi in LNCaP and 22Rv1 cell lines, they suppressed the expression of this lncRNA and identified proliferation and invasiveness of the cells were significantly reduced. In addition, their result indicated the significant association of this lncRNA with clinical features of patients with PCa such as PSA, metastasis, Gleason score, and recurrence-free survival (17).

#### Suppressive lncRNAs in PCa

Some of the identified lncRNAs are tumor-suppressive which show reduced expression in PCa cell and decrease the proliferation and migration of PCa cells through different molecular pathways. Some main suppressive lncRNAs will be discussed as follows. Table II presents the further suppressive lncRNAs with their summary of functions. BDNF-AS (brain-derived neurotrophic factor antisense), a naturally-occurring RNA antisense against BDNF, is downregulated in cancers such as retinoblastoma and lung cancer (46, 47). It was also downregulated in PSA-positive, PSA-negative PCa cell lines as well as PCa human tissue. Further investigation revealed that increasing the expression of this lncRNA using lentivirus-mediated BDNF-AS suppressed PCa cell development, invasion, and proliferation in two aforementioned PCa cell lines. This study also suggested the overexpression of this lncRNA could be considered as a potential method for therapeutic drug for PCa (48). GAS5 (Growth Arrest Specific 5) gene encodes a snoRNA from its intron and a lncRNA from its exonic sequence. The increased level of this lncRNA promotes apoptosis and inhibits the anti-apoptotic abilities of glucocorticoids by binding to the DNA-binding domain of glucocorticoids (49).

A study by Pickard and colleagues using GAS5-encoding plasmids or GAS5 siRNAs in PCa cell lines showed that the low expression of this lncRNA is significantly associated with increased apoptosis and decreased survival rate in PCa (50). In addition, a study found that this lncRNA targets mir-103 leading to inactivation of PI3KAKT-mTOR signaling pathway, low PCa cell growth, and proliferation (51). A single study reported that IGF2-AS (insulin growth factor 2 antisense) lncRNA was downregulated in PCa cell line and human PCa tissues. Lentivirus-induced IGF2AS overexpression decreased xenograft development in vivo, invasion, and proliferation of PCa cells in vitro. Through inverse regulation of IGF2, this lncRNA acted as an epigenetic tumor suppressor in PCa (16). LncRNA FENDRR (FOXF1 Adjacent Non-Coding Developmental Regulatory RNA) is located at 16q24.1. This lncRNA has a fundamental role in modifying chromatin by interacting with Trithorax group/MLL protein complexes (TrxG/MLL) and polycomb repressive complex 2 (PRC2) (52, 53). Zhang and colleagues identified that this lncRNA can also act as ceRNA for miR-18a-5p which upregulates the RUNX1 expression, resulting in decreased progression of PCa cell. Their study also indicated that there was a negative correlation between this lncRNA and the prognosis of PCa (54).

**Table 2:** Other* known suppressive lncRNAs involved in PCa


**lncRNA**	**Genomic location**	**Summary of functions**	**Reference**
**DRAIC**	15q23	Knock down of this lncRNA decreases cell proliferation. It decreases the transformation of cuboidal epithelial cells to fibroblast-like. In addition, it decreases cellular migration	(55)
**LncRNA625**	15p13	Downregulated in PC3 cell line compared to normal cells. It targets miR-432 and decrease its expression, and regulates Wnt/β-catenin pathway. The overexpression of this lncRNA prevents cell growth, induces cell cycle arrest at the G1/S phase and apoptosis in PC3 cells	(56)
**LOC284454**	19p13.13	Downregulated in PCa samples	(57)
**LINC00844**	10q21.1	Induces AR binding to the chromatin and regulates androgen-regulated gene transcription. It activates the expression of NDRG1, which is fundamental metastasis suppressor. Furthermore, it prevents PCa progression and invasion	(58)
**PCAT29**	15q23	Regulated by FOX1 and AR. It suppresses PCa migration and metastasis	(55)
**PCAT14**	22q11.23	Down regulation of this lncRNA is associated with metastatic progression and Gleason score	(59)
**MEG3**	14q32.2	Its expression is low in PCa cells compared to normal cells. It decreases the expression of Bcl-2, increases BAX and activates caspase3, leading to inhibition of intrinsic cell survival pathway. It also inhibits cyclinD1and induces cell cycle arrest in G0/G1 phase	(60)
*Refers to lncRNAs which are not mentioned in the main text Abbreviations: AR: Androgen receptor, NDRG1: N-MYC downstream-regulated gene-1			

### LncRNAs in the discrimination of BPH from PCa

BPH, another common prostate-associated disease, is non-cancerous enlargement of prostate characterized by over-proliferation of stromal and epithelial cells of the transition zone (61). It affects About 50% of males in the age range of 51-60 yr and reaches up to 70% among the age range of 61-70 (62). The measurement of prostate-specific antigen (PSA) has been used widely for screening PCa; however, its level can be increased by BPH as well (63). Therefore, using PSA is controversially debatable (64), and finding biomarkers that can exactly determine PCa from BPH would help clinicians to use proper therapeutic methods for these diseases. Several studies reported that because of tissue-specific expression pattern of lncRNAs, they could be considered as biomarkers to efficiently discriminate BPH from PCa. A study by Bayat and co-authors investigating four lncRNAs, Prcat17.3, Prcat38, Prcat47, and Cat2184.4, revealed low expression of lncRNA Cat2184.4 in PCa samples compared to BPH ones. In contrast, lncRNAs PRCAT17.3 and PRCAT38 showed significant upregulation in PCa samples compared to BPH ones. In addition, lncRNA PRCAT47 showed increased expression in PCa but not statistically significant compared to BPH samples. They concluded that this lncRNA can be considered as potential biomarkers to differentiate BPH from PCa (65).

Another study investigated the expression pattern of exosomal circulating lncRNAs in PCa, BPH and normal samples (66). It is worth mentioning that exosome is vesicle with 40-100 nm diameter, which has fundamental roles in the cell-to-cell communication and cell signaling by binding to their receptor on cells. Furthermore, they can carry a wide variety of molecules such as proteins, miRNAs, and lncRNAs. This structure keeps aforementioned molecules safe from degradation (67). This study revealed that exosomal lncRNA SAP30L-AS1 (SAP30L Antisense RNA 1 (Head To Head)) were significantly upregulated in BPH. However, their result showed that another exosomal lncRNA SChLAP1 (SWI/SNF Complex Antagonist Associated with Prostate Cancer 1) were upregulated in PCa samples compared to BPH and normal ones. The latter one is more useful as biomarker for discriminating BPH from PCa when PSA concentration is in a gray zone (66). lncRNA-p21 is another exosomal lncRNA which its expression pattern had been investigated in urine samples from patients with BPH and PCa. It is found in high level in urine samples of patients with PCa compared to samples from BPH patients (68).

### Prostatitis

Prostatitis is inflammation of the prostate gland that has important roles in the male reproductive system. Although it is amendable, its main pathophysiology and treatment in male infertility are still unknown. It also can affect the assisted reproduction; therefore, finding the main pathogenesis of prostatitis and its negative impact on sperm quantity and quality including apoptosis and DNA integrity is indispensable (69). A recent study by Xu and colleagues examined the association of lncRNA GAS5 (growth arrest-specific transcript 5), located at 1q25.1, with chronic non-bacterial prostatitis (CNP). Their study revealed that the expression of this lncRNA was decreased in prostatitis tissues. In addition, they found that this lncRNA prevented cell proliferation in prostatitis by downregulation of COX2 expression, an enzyme producing prostaglandins. Their study in the GAS5-overexpressed CNP rat model showed the overexpression of this lncRNA decreased prostate volume, inflammatory cells count, and locomotion score. Therefore, the overexpression of lncRNA can reduce the injury of CNP in vivo (4).

### Testicular cancer

Although TCa is generally a rare form of cancer, with the annual incidence of approximately 1%, it is the most prevalent cancer among men with the age range of 14 to 44 years. Testicular germ cell tumors (TGCT) represent nearly 98% of TCa, while the remaining comprise stromal tumors including Sertoli cell tumors, Leydig cell tumors, and other more poorly defined histologic types (70, 71). Newly identified TC are responsive to treatment; however, therapeutic outcomes of theses tumors in high stages are not sufficient (72, 73). Therefore, finding molecular pathways and their regulatory elements involved in the progression and growth of TCa cells such as lncRNA would lead to the identification of the best treatment for TCa. A recent study used NCCIT cell lines (Human testicular embryonic carcinoma cells), a pluripotent extragonadal germ cell tumor cell line, to determine the effect of lncRNA Gm2044 on the growth and proliferation of TCa cells. It revealed that the overexpression of this lncRNA prohibited cell proliferation in vitro. In addition, its results showed this effect was mediated by the miR-202-Rbfox2 pathway (74). LncRNA OIP5-AS1 (Opa-interacting protein 5 antisense RNA 1), located at 15q15.1, is another lncRNA that was shown to be overexpressed and promote cell proliferation in many cancers (75). This lncRNA is highly overexpressed in TGCT compared to other many cancers such as thyroid, prostate, pheochromocytoma, and paraganglioma (76). Based on the study by Rezaie and co-authors lncRNA LINC-ROR (Long Intergenic Non-Protein Coding RNA, Regulator Of Reprogramming) was highly expressed in testicular tumor tissues compared to control cell lines (NT2) (77). This lncRNA is located at 18q21 and is involved in embryonic stem cells (ESC) maintenance (78). Moreover, this lncRNA interacts with heterogeneous nuclear ribonucleoprotein I and suppresses p53 activated by DNA damage (79).

### Varicocele

Varicocele, a main abnormality contributing to male infertility, is an abnormal dilation of pampiniform venous plexus in the scrotum and is prevalent among 20% of adult and adolescent male (80). It causes increased oxidative stress, apoptosis, venous pressure, and temperature resulting in testicular and sperm damage (81). Although many genetics factors such as chromosome alteration and epigenetic changes have been identified to be associated with Varicocele, however, its main molecular mechanism is still unknown (82). lncRNAs as a functional modulator of biological processes may have important roles in the pathogenesis of the disease. The lncRNA gadd7 (growth arrested DNA-damage inducible gene 7), a 754-nt polyadenylated lncRNA, is overexpressed after DNA damage and growth arrest signal. Its overexpression is associated with suppressed cell growth (83, 84). Furthermore, it is the modulator of endoplasmic reticulum stress and lipid-induced oxidative (85). A single study analyzed the expression level of this lncRNA in the ejaculated spermatozoa of patients with Varicocele and found that the expression level lncRNA gadd7 is negatively associated with sperm count. It also used mouse germ cell lines GC-1 and GC-2 transfected with either pcDNA3.1-gadd7 or negative control plasmid to investigate the effect of this lncRNA overexpression on cell features. Its result indicated the overexpression of this lncRNA suppressed cell proliferation and increased cell apoptosis. In addition, the study showed that gadd7 induced by stress can cause cell death through upregulation of Bax and downregulation of Bcl2, which are pro-apoptotic and anti-apoptotic regulators, respectively (86).

### Sperm abnormalities

Male infertility can also be ascribed to uniform testicular maturation arrest (MA) and different types of sperm abnormalities such as oligozoospermia, non-obstructive azoospermia, AZS, and teratozoospermia (87). Spermatogenesis is a complex process regulated by different genes, proteins, and transcriptional network including ncRNAs. Based on the wide ability of lncRNA in proliferation, differentiation, and self-renewal of SSC, they have fundamental roles in spermatogenesis regulation (88). LncRNA HOTAIR (HOX Transcript Antisense Intergenic RNA), located at 12q13.13, is one of the well-studied lncRNA in many diseases and involved in chromatin regulation by binding to Polycomb repressive complex 2 (PRC2) (89). In addition, it plays a key role in epigenetic regulation by interacting with the lysine-specific demethylase 1 (LSD1), Enhancer of Zeste homolog 2 (EZH2),and methyltransferase specific to histone 3 lysine 27 (90). Zhang and colleagues investigated the expression of this lncRNA in the samples from patients with AZS and oligoasthenozoospermia, and found it had a low expression in AZS and oligoasthenozoospermia compared to normal samples. Furthermore, their results indicated that the low expression of HOTAIR is associated with low expression NRF2 (Nuclear factor erythroid 2-related factor 2) gene. HOTAIR is responsible for histone H4 acetylation in the NRF2 gene promoter, which leads to its activation (91). Since the expression level of NRF2 is associated with sperm quality and antioxidant gene expression (92), HOTAIR may protect spermatozoa against antioxidant activity (91). Another study analyzed the expression profile of lncRNA in AZS and normal sperm samples. The gene ontology and pathway analysis revealed that differentially expressed lncRNA in AZS and normal samples were related to sperm function and spermatogenesis. Moreover, among all identified differentially expressed lncRNAs, the expression level of three lncRNA including lnc32058, lnc09522, and lnc98487 were significantly correlated with sperm motility (93).

## 4. Conclusion

In conclusion, this review emphasizes the identified lncRNAs and their functions in male reproductive disorders including PCa, BPH, prostatitis, TCa, varicocele, and sperm abnormalities. With the advent of state-of-the-art molecular and genomics techniques such as high-throughput sequencing, thousands of lncRNA have been identified in susceptibility to different diseases. However, their main mechanisms and molecular pathways are still unknown and need more functional and in vitro studies. Identification of differentially expressed lncRNA in each disease can pave the way toward developing unique biomarker, approaches to treat diseases, as well as increase efficiency of assisted reproductive technologies. In addition, lncRNA can be considered as precious indicator of sperm quality.

##  Conflict of Interest

The authors declare that there is no conflict of interest.

## References

[1] Agarwal A, Mulgund A, Hamada A, Chyatte MR. A unique view on male infertility around the globe. *Reprod Biol Endocrinol* 2015; 13: 37. 10.1186/s12958-015-0032-1 PMC4424520 25928197

[2] Cooper TG, Noonan E, Von Eckardstein S, Auger J, Baker HW, Behre HM, et al. World Health Organization reference values for human semen characteristics. *Hum Reprod Update *2010; 16: 231–245. 10.1093/humupd/dmp048 19934213

[3] Liu J, Cheng G, Yang H, Deng X, Qin Ch, Hua L, et al. Reciprocal regulation of long noncoding RNAs THBS4-003 and THBS4 control migration and invasion in prostate cancer cell lines. *Mol Med Rep* 2016; 14: 1451–1458. 10.3892/mmr.2016.5443 PMC4940078 27357608

[4] Xu X, Hou J, Lv J, Huang Y, Pu J, Wang L. Overexpression of lncRNA GAS5 suppresses prostatic epithelial cell proliferation by regulating COX-2 in chronic non-bacterial prostatitis. *Cell Cycle* 2019; 18: 923–931. 10.1080/15384101.2019.1593644 PMC6527275 30892130

[5] Khademi Bami M, Dehghan Tezerjani M, Montazeri F, Ashrafzadeh Mehrjardi HR, Ghasemi-Esmailabad S, Sheikhha MH, et al. Tumor necrosis factor alpha-308 G/A single nucleotide polymorphism and risk of sperm abnormalities in Iranian males. *Int J Fertil Steril* 2017; 11: 112–116. 10.22074/ijfs.2017.4830 PMC5347448 28670429

[6] Ashrafzadeh HR, Nazari T, Dehghan Tezerjani M, Khademi Bami M, Ghasemi-Esmailabad S, Ghasemi N. Frequency of TNFR1 36 A/G gene polymorphism in azoospermic infertile men: A case-control study. *Int J Reprod BioMed* 2017; 15: 521–526. PMC5653914 29082371

[7] Hangauer MJ, Vaughn IW, McManus MT. Pervasive transcription of the human genome produces thousands of previously unidentified long intergenic noncoding RNAs. *Plos Genet* 2013; 9: e1003569. 1–13. 10.1371/journal.pgen.1003569 PMC3688513 23818866

[8] Morceau F, Chateauvieux S, Gaigneaux A, Dicato M, Diederich M. Long and short non-coding RNAs as regulators of hematopoietic differentiation. *Int J Mol Sci *2013; 14: 14744–14770. 10.3390/ijms140714744 PMC3742271 23860209

[9] Kadali VN, Chandran Sh, Murthy S. Long Non-coding RNAs and their “orchestration” in cancers. *Journal of Applied Biology & Biotechnology* 2018; 6: 57–60.

[10] Tate PH, Bird AP. Effects of DNA methylation on DNA-binding proteins and gene expression. *Curr Opin Genet Dev *1993; 3: 226–231. 10.1016/0959-437x(93)90027-m 8504247

[11] Nathan D, Sterner DE, Berger SL. Histone modifications: Now summoning sumoylation. *Proc Natl Acad Sci USA *2003; 100: 13118–13120. 10.1073/pnas.2436173100 PMC263724 14597707

[12] Engreitz JM, Haines JE, Perez EM, Munson G, Chen J, Kane M, et al. Local regulation of gene expression by lncRNA promoters, transcription and splicing. *Nature* 2016; 539: 452–455. 10.1038/nature20149 PMC6853796 27783602

[13] Evans JR, Feng FY, Chinnaiyan AM. The bright side of dark matter: lncRNAs in cancer. *J Clin Invest *2016; 126: 2775–2782. 10.1172/JCI84421 PMC4966302 27479746

[14] Mukherjee A, Koli S, Reddy K. Regulatory non-coding transcripts in spermatogenesis: shedding light on `dark matter'. *Andrology* 2014; 2: 360–369. 10.1111/j.2047-2927.2014.00183.x 24519965

[15] Bray F, Ferlay J, Soerjomataram I, Siegel RL, Torre LA, Jemal A. Global cancer statistics 2018: GLOBOCAN estimates of incidence and mortality worldwide for 36 cancers in 185 countries. *CA Cancer J Clin *2018; 68: 394–424. 10.3322/caac.21492 30207593

[16] Chen Q, Sun T, Wang F, Gong B, Xie W, Ma M, et al. Long noncoding RNA IGF2AS is acting as an epigenetic tumor suppressor in human prostate cancer. *Urology* 2019; 124: 310–318. 10.1016/j.urology.2018.11.002 30423304

[17] Jiang CY, Gao Y, Wang XJ, Ruan Y, Bei XY, Wang XH, et al. Long non-coding RNA lnc-MX1-1 is associated with poor clinical features and promotes cellular proliferation and invasiveness in prostate cancer. *Biochem Biophys Res Commun* 2016; 470: 721–727. 10.1016/j.bbrc.2016.01.056 26797523

[18] Qin X, Zhu W, Lu A, Wang G, Ye X, Weng G. Long non-coding RNA SAP30L-AS1 promotes prostate cancer growth through repressing SAP30L. *Gene* 2019; 690: 120–128. 10.1016/j.gene.2018.12.047 30599235

[19] Zhao R, Sun F, Bei X, Wang X, Zhu Y, Jiang C, et al. Upregulation of the long non-coding RNA FALEC promotes proliferation and migration of prostate cancer cell lines and predicts prognosis of PCa patients. *Prostate* 2017; 77: 1107–1117. 10.1002/pros.23367 28585762

[20] Wang J, Yang X, Li R, Wang L, Gu Y, Zhao Y, et al. Long non-coding RNA MYU promotes prostate cancer proliferation by mediating the miR-184/c-Myc axis. *Oncol Rep* 2018; 40: 2814–2825. 10.3892/or.2018.6661 30132573

[21] Yang X, Wang L, Li R, Zhao Y, Gu Y, Liu S, et al. The long non-coding RNA PCSEAT exhibits an oncogenic property in prostate cancer and functions as a competing endogenous RNA that associates with EZH2. *Biochem Biophys Res Commun *2018; 502: 262–268. 10.1016/j.bbrc.2018.05.157 29803673

[22] Chen Y, Gu M, Liu Ch, Wan X, Shi Q, Chen Q, et al. Long noncoding RNA FOXC2-AS1 facilitates the proliferation and progression of prostate cancer via targeting miR-1253/EZH2. *Gene* 2019; 686: 37–42. 10.1016/j.gene.2018.10.085 30389560

[23] Beaver LM, Kuintzle R, Buchanan A, Wiley MW, Glasser ST, Wong CP, et al. Long noncoding RNAs and sulforaphane: a target for chemoprevention and suppression of prostate cancer. *J Nutr Biochem* 2017; 42: 72–83. 10.1016/j.jnutbio.2017.01.001 PMC5360475 28131897

[24] Wang J, Cheng G, Li X, Pan Y, Qin Ch, Yang H, et al. Overexpression of long non-coding RNA LOC400891 promotes tumor progression and poor prognosis in prostate cancer. *Tumor Biol *2016; 37: 9603–9613. 10.1007/s13277-016-4847-y 26797783

[25] Liu T, Chi H, Chen J, Chen Ch, Huang Y, Xi H, et al. Curcumin suppresses proliferation and in vitro invasion of human prostate cancer stem cells by ceRNA effect of miR-145 and lncRNA-ROR. *Gene* 2017; 631: 29–38. 10.1016/j.gene.2017.08.008 28843521

[26] Lemos AEG, Ferreira LB, Batoreu NM, de Freitas PP, Bonamino MH, Gimba ERP. PCA3 long noncoding RNA modulates the expression of key cancer-related genes in LNCaP prostate cancer cells. *Tumour Biol* 2016; 37: 11339–11348. 10.1007/s13277-016-5012-3 26960690

[27] Lee B, Mazar J, Aftab MN, Qi F, Shelley J, Li JL, et al. Long noncoding RNAs as putative biomarkers for prostate cancer detection. *J Mol Diagn *2014; 16: 615–626. 10.1016/j.jmoldx.2014.06.009 PMC4210464 25307116

[28] Zhang Ch, Liu Ch, Wu J, Zheng Y, Xu H, Cheng G, et al. Upregulation of long noncoding RNA LOC440040 promotes tumor progression and predicts poor prognosis in patients with prostate cancer. *Onco Targets Ther *2017; 10: 4945–4954. 10.2147/OTT.S138354 PMC5644598 29066914

[29] Su W, Xu M, Chen X, Chen N, Gong J, Nie L, et al. Long noncoding RNA ZEB1-AS1 epigenetically regulates the expressions of ZEB1 and downstream molecules in prostate cancer. *Molecular Cancer *2017; 16: 142. 10.1186/s12943-017-0711-y PMC5568204 28830551

[30] Orfanelli U, Jachetti E, Chiacchiera F, Grioni M, Brambilla P, Briganti A, et al. Antisense transcription at the TRPM2 locus as a novel prognostic marker and therapeutic target in prostate cancer. *Oncogene* 2015; 34: 2094–2102. 10.1038/onc.2014.144 24931166

[31] Lavorgna G, Chiacchiera F, Briganti A, Montorsi F, Pasini D, Salonia A. Expression-profiling of apoptosis induced by ablation of the long ncRNA TRPM2-AS in prostate cancer cell. *Genom Data *2015; 3: 4–5. 10.1016/j.gdata.2014.10.020 PMC4535459 26484139

[32] Prensner JR, Iyer MK, Balbin OA, Dhanasekaran SM, Cao Q, Brenner JC, et al. Transcriptome sequencing across a prostate cancer cohort identifies PCAT-1, an unannotated lincRNA implicated in disease progression. *Nat Biotechnol* 2011; 29: 742–749. 10.1038/nbt.1914 PMC3152676 21804560

[33] Prensner JR, Chen W, Iyer MK, Cao Q, Ma T, Han S, et al. PCAT-1, a long noncoding RNA, regulates BRCA2 and controls homologous recombination in cancer. *Cancer Res* 2014; 74: 1651–1660. 10.1158/0008-5472.CAN-13-3159 PMC4009928 24473064

[34] Prensner JR, Chen W, Han S, Iyer MK, Cao Q, Kothari V, et al. The long non-coding RNA PCAT-1 promotes prostate cancer cell proliferation through cMyc. *Neoplasia* 2014; 16: 900–908. 10.1016/j.neo.2014.09.001 PMC4240923 25425964

[35] Ylip A, Kivinummi K, Kohvakka A, Annala M, Latonen L, Scaravilli M, et al. Transcriptome sequencing reveals PCAT5 as a novel ERG-regulated long noncoding RNA in prostate cancer. *Cancer Res *2015; 75: 4026–4031. 10.1158/0008-5472.CAN-15-0217 26282172

[36] Misawa A, Takayama KI, Fujimura T, Homma Y, Suzuki Y, Inoue S. Androgen-induced lncRNA POTEF-AS1 regulates apoptosis-related pathway to facilitate cell survival in prostate cancer cells. *Cancer Sci* 2017; 108: 373–379. 10.1111/cas.13151 PMC5378265 28032932

[37] Gu P, Chen X, Xie R, Han J, Xie W, Wang B, et al. lncRNA HOXD-AS1 regulates proliferation and chemo-resistance of castration-resistant prostate cancer via recruiting WDR5. *Mol Ther *2017; 25: 1959–1973. 10.1016/j.ymthe.2017.04.016 PMC5542640 28487115

[38] Li J, Zhang Zh, Xiong L, Guo Ch, Jiang T, Zeng L, et al. SNHG1 lncRNA negatively regulates miR-199a-3p to enhance CDK7 expression and promote cell proliferation in prostate cancer. *Biochem Biophys Res Commun* 2017; 487: 146–152. 10.1016/j.bbrc.2017.03.169 28400279

[39] Misawa A, Takayama KI, Urano T, Inoue S. Androgen-induced long noncoding RNA (lncRNA) SOCS2-AS1 promotes cell growth and inhibits apoptosis in prostate cancer cells.* J Biol Chem *2016; 291: 17861–17880. 10.1074/jbc.M116.718536 PMC5016176 27342777

[40] Takayama KI, Horie-Inoue K, Katayama Sh, Suzuki T, Tsutsumi Sh, Ikeda K, et al. Androgen-responsive long noncoding RNA CTBP1-AS promotes prostate cancer. *EMBO J* 2013; 32: 1665–1680. 10.1038/emboj.2013.99 PMC3680743 23644382

[41] Liu HT, Fang L, Cheng YX, Sun Q. LncRNA PVT1 regulates prostate cancer cell growth by inducing the methylation of miR-146a. *Cancer Med* 2016; 5: 3512–3519. 10.1002/cam4.900 PMC5224852 27794184

[42] Yang J, Li C, Mudd A, Gu X. LncRNA PVT1 predicts prognosis and regulates tumor growth in prostate cancer. *Biosci Biotechnol Biochem *2017; 81: 2301–2306. 10.1080/09168451.2017.1387048 29050519

[43] Zhu Y, Ren Sh, Jing T, Cai X, Liu Y, Wang F, et al. Clinical utility of a novel urine-based gene fusion TTTY15-USP9Y in predicting prostate biopsy outcome. *Urol Oncol* 2015; 33: 384. e9–e20. 10.1016/j.urolonc.2015.01.019 26008593

[44] Ren Sh, Peng Zh, Mao JH, Yu Y, Yin Ch, Gao X, et al. RNA-seq analysis of prostate cancer in the Chinese population identifies recurrent gene fusions, cancer-associated long noncoding RNAs and aberrant alternative splicings. *Cell Res *2012; 22: 806–821. 10.1038/cr.2012.30 PMC3343650 22349460

[45] Yao J, Kong D, Ye C, Chen R, Li L, Zeng T, et al. The long noncoding RNA TTTY15, which is located on the Y chromosome, promotes prostate cancer progression by sponging let-7. *Eur Urol* 2019; 76: 315–326. 10.1016/j.eururo.2018.11.012 30527798

[46] Shang W, Yang Y, Zhang J, Wu Q. Long noncoding RNA BDNF-AS is a potential biomarker and regulates cancer development in human retinoblastoma. *Biochem Biophys Res Commun* 2018; 497: 1142–1148. 10.1016/j.bbrc.2017.01.134 28131827

[47] Shen M, Xu Zh, Jiang K, Xu W, Chen Y, Xu ZH. Long noncoding nature brain-derived neurotrophic factor antisense is associated with poor prognosis and functional regulation in non-small cell lung caner. *Tumour Biol* 2017; 39: 1010428317695948. 1–9. 10.1177/1010428317695948 28459374

[48] Li W, Dou Zh, We Sh, Zhu Zh, Pan D, Jia Zh, et al. Long noncoding RNA BDNF-AS is associated with clinical outcomes and has functional role in human prostate cancer. *Biomed Pharmacother* 2018; 102: 1105–1110. 10.1016/j.biopha.2018.03.118 29710528

[49] Kino T, Hurt DE, Ichijo T, Nader N, Chrousos GP. Noncoding RNA gas5 is a growth arrest-and starvation-associated repressor of the glucocorticoid receptor. *Sci Signal* 2010; 3: ra8. 10.1126/scisignal.2000568 PMC2819218 20124551

[50] Pickard MR, Mourtada-Maarabouni M, Williams GT. Long non-coding RNA GAS5 regulates apoptosis in prostate cancer cell lines. *Biochim Biophys Acta *2013; 1832: 1613–1623. 10.1016/j.bbadis.2013.05.005 23676682

[51] Xue D, Zhou C, Lu H, Xu R, Xu X, He X. LncRNA GAS5 inhibits proliferation and progression of prostate cancer by targeting miR-103 through AKT/mTOR signaling pathway. *Tumour Biol* 2016; 37: 16187–16197. 10.1007/s13277-016-5429-8 27743383

[52] Schuettengruber B, Chourrout D, Vervoort M, Leblanc B, Cavalli G. Genome regulation by polycomb and trithorax proteins. *Cell* 2007; 128: 735–745. 10.1016/j.cell.2007.02.009 17320510

[53] Khalil AM, Guttman M, Huarte M, Garber M, Raj A, Morales DR, et al. Many human large intergenic noncoding RNAs associate with chromatin-modifying complexes and affect gene expression. *Proc Natl Acad Sci* USA 2009; 106: 11667–11672. 10.1073/pnas.0904715106 PMC2704857 19571010

[54] Zhang G, Han G, Zhang X, Yu Q, Li Z, Li Zh, et al. Long non-coding RNA FENDRR reduces prostate cancer malignancy by competitively binding miR-18a-5p with RUNX1. *Biomarkers* 2018; 23: 435–445. 10.1080/1354750X.2018.1443509 29465000

[55] Sakurai K, Reon BJ, Anaya J, Dutta A. The lncRNA DRAIC/PCAT29 locus constitutes a tumor-suppressive nexus. *Mol Cancer Res* 2015; 13: 828–838. 10.1158/1541-7786.MCR-15-0016-T PMC4456356 25700553

[56] Li JB, Liu F, Zhang BP, Bai WK, Cheng W, Zhang YH, et al. LncRNA625 modulates prostate cancer cells proliferation and apoptosis through regulating the Wnt/β-catenin pathway by targeting miR-432. *Eur Rev Med Pharmacol Sci* 2017; 21: 2586–2595. 28678327

[57] Das M, Renganathan A, Dighe SN, Bhaduri U, Shettar A, Mukherjee G, et al. DDX5/p68 associated lncRNA LOC284454 is differentially expressed in human cancers and modulates gene expression. *RNA Biol *2018; 15: 214–230. 10.1080/15476286.2017.1397261 PMC5798960 29227193

[58] Lingadahalli Sh, Jadhao S, Sung YY, Chen M, Hu L, Chen X, et al. Novel lncRNA LINC00844 regulates prostate cancer cell migration and invasion through AR signaling. *Mol Cancer Res* 2018; 16: 1865–1878. 10.1158/1541-7786.MCR-18-0087 30115758

[59] White NM, Zhao SG, Zhang J, Rozycki EB, Dang HX, McFadden SD, et al. Multi-institutional analysis shows that low PCAT-14 expression associates with poor outcomes in prostate cancer. *Eur Urol *2017; 71: 257–266. 10.1016/j.eururo.2016.07.012 27460352

[60] Luo G, Wang M, Wu X, Tao D, Xiao X, Wang L, et al. Long non-coding RNA MEG3 inhibits cell proliferation and induces apoptosis in prostate cancer. *Cell Physiol Biochem* 2015; 37: 2209–2220. 10.1159/000438577 26610246

[61] Descazeaud A, Rubin MA, Hofer M, Setlur S, Nikolaief N, Vacherot F, et al. BPH gene expression profile associated to prostate gland volume. *Diagn Mol Pathol* 2008; 17: 207–213. 10.1097/PDM.0b013e31816f6352 PMC2822796 18936709

[62] McVary KT. BPH: epidemiology and comorbidities. *Am J Manag Care* 2006; 12: S122–S128. 16613526

[63] Armitage TG, Cooper EH, Newling DW, Robinson MR, Appleyard I. The value of the measurement of serum prostate specific antigen in patients with benign prostatic hyperplasia and untreated prostate cancer. *Br J Urol* 1988; 62: 584–589. 10.1111/j.1464-410x.1988.tb04431.x 2464397

[64] Bokhorst LP, Bangma ChH, van Leenders GJ, Lous JJ, Moss SM, Schröder FH, et al. Prostate-specific antigen-based prostate cancer screening: Reduction of prostate cancer mortality after correction for nonattendance and contamination in the rotterdam section of the European randomized study of screening for prostate cancer. *Eur Urol *2014; 65: 329–336. 10.1016/j.eururo.2013.08.005 23954085

[65] Bayat H, Narouie B, Ziaee SAM, Mowla SJ. Two long non-coding RNAs, Prcat17. 3 and Prcat38, could efficiently discriminate benign prostate hyperplasia from prostate cancer. *Prostate* 2018; 78: 812–818. 10.1002/pros.23538 29671889

[66] Wang YH, Ji J, Wang BC, Chen H, Yang ZhH, Wang K, et al. Tumor-derived exosomal long noncoding RNAs as promising diagnostic biomarkers for prostate cancer. *Cell Physiol Biochem* 2018; 46: 532–545. 10.1159/000488620 29614511

[67] Lässer C. Identification and analysis of circulating exosomal microRNA in human body fluids. *Methods Mol Biol* 2013; 1024: 109–128. 10.1007/978-1-62703-453-1_9 23719946

[68] Işin M, Uysaler E, Özgür E, Köseoğlu H, Şanlı Ö, Yücel ÖB, et al. Exosomal lncRNA-p21 levels may help to distinguish prostate cancer from benign disease. *Front Genet* 2015; 6: 168. 10.3389/fgene.2015.00168 PMC4422020 25999983

[69] Alshahrani S, McGill J, Agarwal A. Prostatitis and male infertility. *J Reprod Immunol* 2013; 100: 30–36. 10.1016/j.jri.2013.05.004 23938147

[70] McGlynn KA, Cook MB. Etiologic factors in testicular germ-cell tumors. *Future Oncol* 2009; 5: 1389–1402. 10.2217/fon.09.116 PMC3000220 19903067

[71] De Toni L, Šabovic I, Cosci I, Ghezzi M, Foresta C, Garolla A. Testicular cancer: Genes, environment, hormones. *Front Endocrinol* 2019; 10: 408. 10.3389/fendo.2019.00408 PMC6626920 31338064

[72] van de Geijn GJM, Hersmus R, Looijenga LH. Recent developments in testicular germ cell tumor research. *Birth Defects Res C* 2009; 87: 96–113. 10.1002/bdrc.20140 19306344

[73] Cost NG, Adibi M, Lubahn JD, Romman A, Raj GV, Sagalowsky AI, et al. Effect of testicular germ cell tumor therapy on renal function. *Urology* 2012; 80: 641–648. 10.1016/j.urology.2012.04.064 22840865

[74] Liang M, Hu CK, He Ch, Zhou J, Liao Y. Upregulated lncRNA Gm2044 inhibits male germ cell development by acting as miR-202 host gene. *Anim Cells Syst* 2019; 23: 128–134. 10.1080/19768354.2019.1591506 PMC6440523 30949400

[75] Sun WL, Kang T, Wang YY, Sun JP, Li Ch, Liu HJ, et al. Long noncoding RNA OIP5-AS1 targets Wnt-7b to affect glioma progression via modulation of miR-410. *Biosci Rep *2019; 39: BSR20180395. 1–11. 10.1042/BSR20180395 PMC6328889 30498093

[76] Arunkumar G, Anand Sh, Raksha P, Dhamodharan Sh, Rao HPS, Subbiah Sh, et al. LncRNA OIP5-AS1 is overexpressed in undifferentiated oral tumors and integrated analysis identifies AS a downstream effector of stemness-associated transcription factors. *Sci Rep* 2018; 8: 7018. 1–13. 10.1038/s41598-018-25451-3 PMC5935738 29728583

[77] Rezaei M, Emadi-Baygi M, Hoffmann MJ, Schulz WA, Nikpour P. Altered expression of LINC-ROR in cancer cell lines and tissues. *Tumor Biol *2016; 37: 1763–1769. 10.1007/s13277-015-3933-x 26314857

[78] Wang Y, Xu Zh, Jiang J, Xu Ch, Kang J, Xiao L, et al. Endogenous miRNA sponge lincRNA-RoR regulates Oct4, Nanog, and Sox2 in human embryonic stem cell self-renewal. *Dev Cell* 2013; 25: 69–80. 10.1016/j.devcel.2013.03.002 23541921

[79] Zhang A, Zhou N, Huang J, Liu Q, Fukuda K, Ma D, et al. The human long non-coding RNA-RoR is a p53 repressor in response to DNA damage. *Cell Res *2013; 23: 340–350. 10.1038/cr.2012.164 PMC3587705 23208419

[80] Weidner W, Pilatz A, Altinkilic B. Andrology: varicocele: an update.* Urologe A* 2010; 49 (Suppl.): 163–165. 10.1007/s00120-010-2374-9 20812044

[81] Fretz PC, Sandlow JI. Varicocele: current concepts in pathophysiology, diagnosis, and treatment. *Urol Clin North Am* 2002; 29: 921–937. 10.1016/s0094-0143(02)00075-7 12516762

[82] Santana VP, Miranda-Furtado CL, de Oliveira-Gennaro FG, Dos Reis RM. Genetics and epigenetics of varicocele pathophysiology: An overview.* J Assist Reprod Genet* 2017; 34: 839–847. 10.1007/s10815-017-0931-5 PMC5476544 28523408

[83] Hollander MC, Alamo I, Fornace Jr AJ. A novel DNA damage-inducible transcript, gadd7, inhibits cell growth, but lacks a protein product. *Nucleic Acids Res* 1996; 24: 1589–1593. 10.1093/nar/24.9.1589 PMC145844 8649973

[84] Fornace AJ, Alamo I, Hollander MC. DNA damage-inducible transcripts in mammalian cells. *Proc Natl Acad Sci USA *1988; 85: 8800–8804. 10.1073/pnas.85.23.8800 PMC282594 3194391

[85] Brookheart RT, Michel CI, Listenberger LL, Ory DS, Schaffer JE. The non-coding RNA gadd7 is a regulator of lipid-induced oxidative and endoplasmic reticulum stress. *J Biol Chem* 2009; 284: 7446–7454. 10.1074/jbc.M806209200 PMC2658040 19150982

[86] Zhao J, Li H, Deng H, Zhu L, Zhou B, Yang M, et al. LncRNA gadd7, increased in varicocele patients, suppresses cell proliferation and promotes cell apoptosis. *Oncotarget* 2018; 9: 5105–5110. 10.18632/oncotarget.23696 PMC5797036 29435165

[87] Hung AJ, King P, Schlegel PN. Uniform testicular maturation arrest: a unique subset of men with nonobstructive azoospermia. *J Urol* 2007; 178: 608–612. 10.1016/j.juro.2007.03.125 17570432

[88] Zhang X, Gao F, Fu J, Zhang P, Wang Y, Zeng X. Systematic identification and characterization of long non-coding RNAs in mouse mature sperm.* PLoS One* 2017; 12: e0173402. 10.1371/journal.pone.0173402 PMC5349675 28291811

[89] Gupta RA, Shah N, Wang KC, Kim J, Horlings HM, Wong DJ, et al. Long non-coding RNA HOTAIR reprograms chromatin state to promote cancer metastasis. *Nature* 2010; 464: 1071–1076. 10.1038/nature08975 PMC3049919 20393566

[90] Tsai MCh, Manor O, Wan Y, Mosammaparast N, Wang JK, Lan F, et al. Long noncoding RNA as modular scaffold of histone modification complexes. *Science* 2010; 329: 689–693. 10.1126/science.1192002 PMC2967777 20616235

[91] Zhang L, Liu Zh, Li X, Zhang P, Wang J, Zhu D, et al. Low long non-coding RNA HOTAIR expression is associated with down-regulation of Nrf2 in the spermatozoa of patients with asthenozoospermia or oligoasthenozoospermia. *Int J Clin Exp Pathol *2015; 8: 14198–14205. PMC4713519 26823733

[92] Chen K, Mai Z, Zhou Y, Gao X, Yu B. Low NRF2 mRNA expression in spermatozoa from men with low sperm motility. *Tohoku J Exp Med *2012; 228: 259–266. 10.1620/tjem.228.259 23089668

[93] Zhang X, Zhang P, Song D, Xiong S, Zhang H, Fu J, et al. Expression profiles and characteristics of human lncRNA in normal and asthenozoospermia sperm. *Biol Reprod* 2018; 100: 982– 993. 10.1093/biolre/ioy253 30517597

